# Nutritional, bioactive compounds content, and antioxidant activity of brown seaweeds from the Red Sea

**DOI:** 10.3389/fnut.2023.1210934

**Published:** 2023-07-26

**Authors:** Mona M. Ismail, Gehan M. El Zokm, José M. Miranda Lopez

**Affiliations:** ^1^Department of Marine Environment, National Institute of Oceanography and Fisheries, Cairo, Egypt; ^2^Laboratorio de Higiene, Inspección y Control de Alimentos (LHICA), Departamento de Química Analí-tica, Nutrición y Bromatología, Universidade de Santiago de Compostela, Lugo, Spain

**Keywords:** antioxidants, Phaeophyta, phenolic compounds, pigments, vitamins, proximate

## Abstract

**Introduction:**

Brown seaweeds are excellent sources of bioactive molecules with a wide range of pharmacological effects, whose content can vary depending on several factors, including the origin and the environment in which the algae grow.

**Methods:**

This study aimed to estimate 19 compounds regarding primary and secondary metabolites of eight brown macroalgal species from a clean Egyptian Red Sea coast. A proximate analysis, pigment, phenolic compounds, and vitamin contents were determined. In addition, the energy content and antioxidant activity were estimated to explore the potential application of algae as functional foods to encourage the species’ commercialization.

**Results:**

Based on the chemical composition, *Polycladia myrica* was the most valuable species, with a comparatively high protein content of 22.54%, lipid content of 5.21%, fucoxanthin content of 3.12 μg/g, β-carotene content of 0.55 mg/100 g, and carbohydrate content of 45.2%. This species also acts as a great source of vitamin C, flavonoids, tannins, phenol content and total antioxidant capacity.

**Discussion:**

The antioxidant activity of the selected algae indicated that its phenol, vitamin and pigment contents were powerful antioxidant compounds based on the structure–activity relationships. This result was verified by the strong correlation in statistical analysis at the 95% confidence level. From a worldwide perspective and based on the obtained results, these brown species may be reinforced as an essential line in future foods.

## Introduction

The global variety of all algae (micro and macro) consists of over 164,000 species, with approximately 9,800 of them being seaweeds, 0.17% of which have been domesticated for commercial exploitation (1, 2). In recent years, the consumption of seaweeds has gained popularity in countries where their consumption was not a traditional custom, both as a food ingredient and for making beverages. Many types of seaweeds are edible by humans and are an important source of proteins, polysaccharides, vitamins and minerals (3–5). In general, their nutritional composition can vary significantly, depending on many factors (6).

Among the three groups of macroalgae, brown seaweeds have received considerable attention because of their numerous bioactive compounds and biological properties. Among bioactive properties, brown seaweeds can cause beneficial effects on improving the microbiota, antioxidant and anti-inflammatory properties, dyslipidemia control, reduced obesity, diabetes control, immunomodulatory, dyslipidemia, hypertension control, diabetes, and some types of cancer (7).

Brown seaweeds have a distinctive and abundant composition of pertinent polysaccharides and bioactive substances that can make up as much as 70% of the tissue with several uses in food, nutraceutical, pharmaceutical, cosmetic, and biopolymer applications (8). Brown seaweeds are characterized by the production of several types of carotenoids, such as fucoxanthin and β-carotene, which have anti-obesity properties and antioxidant activity. Moreover, they are a valuable source of lipophilic antioxidants such as vitamins A (retinoic acid), C (ascorbic acid) and E (tocopherol) (9). Algal vitamins are important not only for biochemical functions and antioxidant activity but also for other health benefits, such as lowering blood pressure (9). Vitamin C is a water-soluble vitamin and is considered an essential micronutrient with antioxidant function that can reduce the risk for stomach cancer (8). Additionally, β-carotene, carotenoids, vitamins E and C contribute to preventing cardiovascular disease and lowering cancer risk (8, 9).

The antioxidant substances contained in algae depend on their exposure to external environmental factors such as salinity, nutrient availability, light, depth at which they grow and seasonality. In addition, they also depend on intrinsic factors such as algal species, age, length and tissue type (6). The main groups of antioxidants in seaweeds are phenolics, polysaccharides, vitamins, and pigments (10, 11). Polyphenol compounds (phenolic acid, flavonoids, and tannins) have been classified as strong antioxidant compounds (3). In this work, eight brown seaweeds were selected for valorization regarding nutritional characteristics and bioactive profiles, all of which are widely grown throughout the Red Sea and relatively underexplored. Such seaweeds were tested as sources of bioactive components with antioxidant properties. This trend is aimed at addressing the issues that the world has encountered because of population growth outpacing food resources. The relationship between the chemical structure and antioxidant activity of the selected compounds was also elucidated.

## Materials and methods

### Collection of macroalgal species

**Figure 1:**
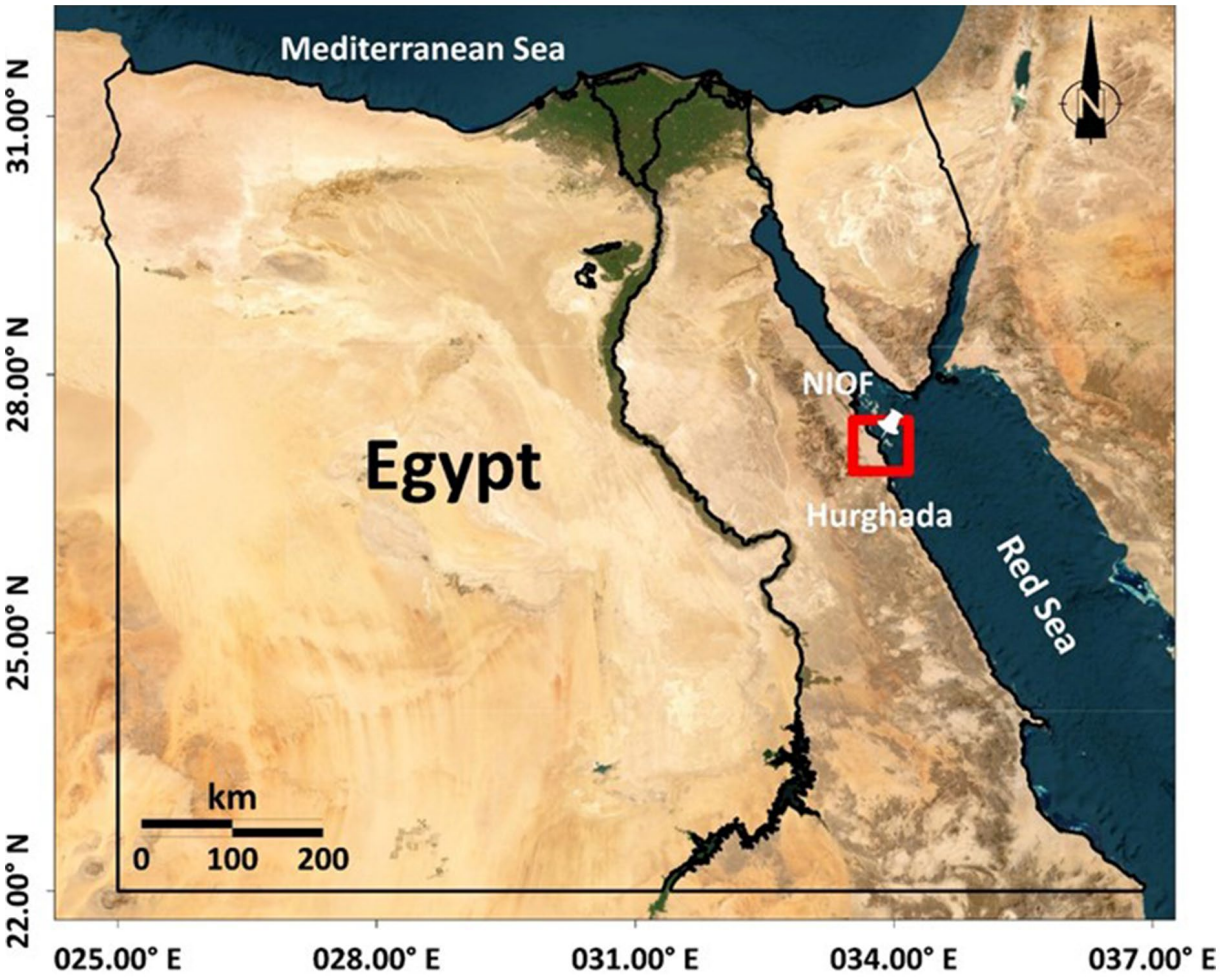
Sampling location.

**Figure 2:**
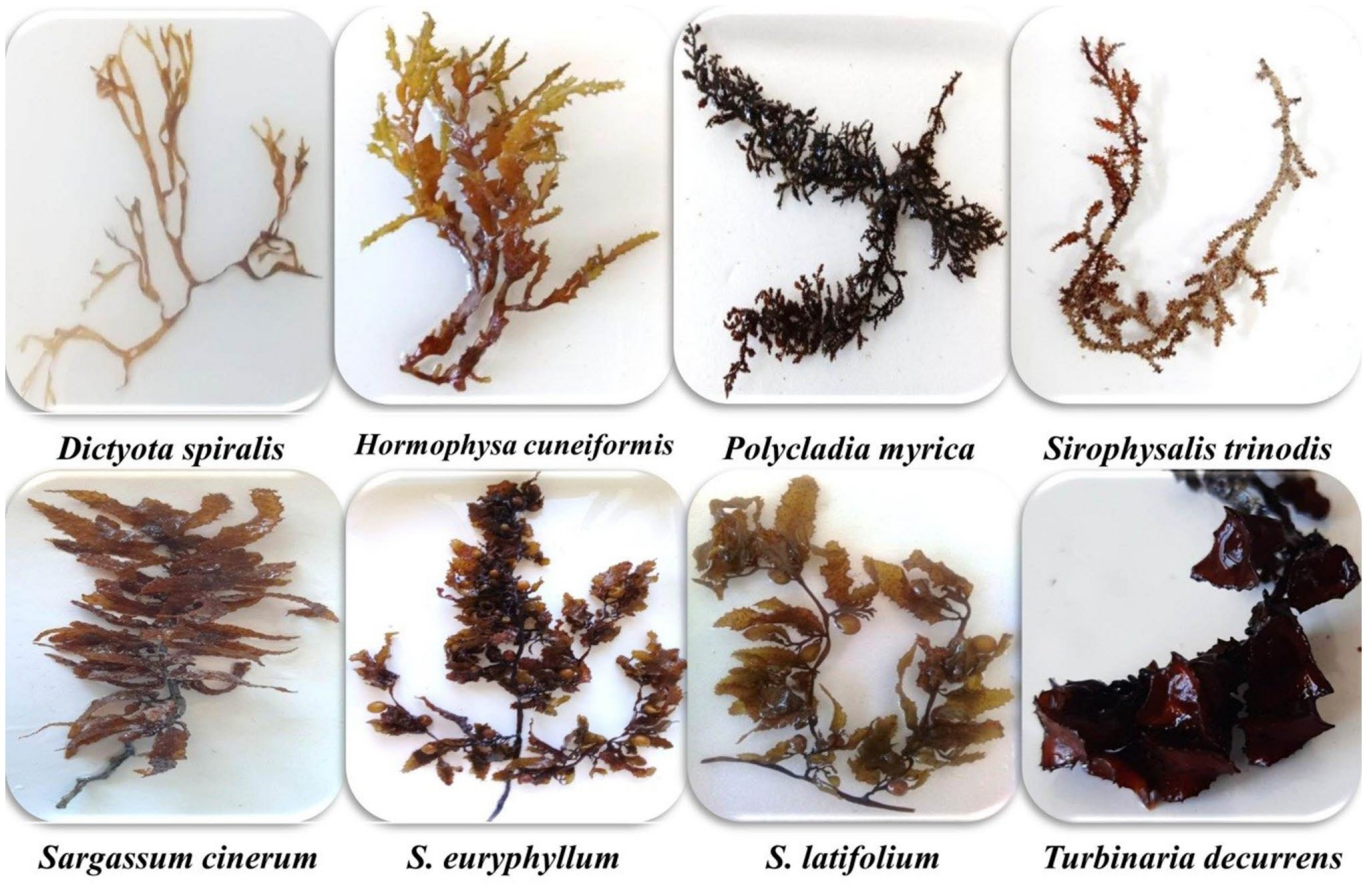
Photo of the collected brown species.

Eight species of brown algae were handpicked collected during the winter months of 2020 in the vicinity of the National Institute of Oceanography and Fisheries, between latitude 27°17′13” N and longitude 33°46′21″ E, located in Hurghada city, Red Sea, Egypt (Figure 1). The collected samples were thoroughly washed with distilled water and, subsequently, cleaned with a soft brush to remove deposits and epiphytes. A portion of the fresh algae was then processed as herbarium specimens. Other fresh samples were preserved in fresh seawater spiked with 5% formalin for taxonomic classification. The other portions were dried at room temperature in a place protected from sunlight, ground to a fine powder, and stored at −20°C until use. The taxonomy of the algal samples was explored using their morphological characteristics (12, 13), and species names were verified according to the Algae Base website (14) (Figure 2). Species collected from the studied sites were identified as *Dictyota spiralis* Montagne, *Hormophysa cuneiformis* (J.F. Gmelin) P.C. Silva, *Polycladia myrica* (S.G. Gmelin) C. Agardh, *Sirophysalis trinodis* (Forsskal) Kützing, *Sargassum cinerum* (J. Agardh), *Sargassum euryphyllum* (Grunow) Tseng & Lu Baoren, Sargassum latifolium (Turner) C. Agardh and *Turbinaria decurrens* (Bory de Saint-Vincent).

### Biochemical analysis

The carbohydrate content was determined using the method described by Dubois et al. (15). The total protein content was quantified in accordance with the method described by Lowry et al. (16). The lipids were determined by Soxhlet extraction on the basis of the Association of Official Analytical Chemists (AOAC) assay (17). These parameters were expressed as ratios (%). The ash content of the algae were determined by muffle incineration at 550°C in accordance with the AOAC method (17). Crude fiber was estimated by acid and alkaline digestion according to AOAC (17) method and expressed as percentage loss in weight on ignition.


$$\mathrm{Crude fiber}=\left[\left(W_{1}-W_{2}\right)/W\right]\times 100.$$

where W_1_ is the weight (g) of the digested sample before incineration, W_2_ is the weight (g) of the digested sample after incineration, and W is the weight (g) of the algal sample taken.

The caloric contents were determined based on the following formula:


$$\begin{array}{l} \mathrm{Calorie Value}\;\left(\mathrm{kcal}/100g\;\mathrm{DW}\right)\\ =9\times \mathrm{Lipid}\;\left(\%\right)+4\times \mathrm{Protein}\;\left(\%\right)+4\times \mathrm{Carbohydrate}\;\left(\%\right) \end{array}$$

Chlorophyll contents “*a*, *c*, and *d*” and carotenoids in the algal acetone extracts (90%) were detected using the technique described by Connan (18). They are estimated using the following formula and expressed as μg/g fresh weight.


$$\begin{array}{l} \mathrm{Chlorophyll}‘a’\;\left(\mu g/g\right)\\ =\frac{\left[\begin{array}{c} -0.3002\times \left(A_{630}-A_{750}\right)-1.75\times \left(A_{647}-A_{750}\right)\\ +11.909\times \left(A_{665}-A_{750}\right) \end{array}\right]}{\mathrm{Weight of the sample}} \end{array}$$

$$\begin{array}{l} \mathrm{Chlorophyll}‘c’\;\left(\mu g/g\right)\\ =\frac{\left[\begin{array}{c} -23.6723\times \left(A_{630}-A_{750}\right)+7.9057\times \left(A_{647}-A_{750}\right)\\ -1.5467\times \left(A_{665}-A_{750}\right) \end{array}\right]}{\mathrm{Weight of the sample}} \end{array}$$

$$\begin{array}{l} \mathrm{Chlorophyll}‘d’\;\left(\mu g/g\right)\\ =\frac{\left[\begin{array}{l} -0.3411\times \left(A_{630}-A_{750}\right)+0.1129\times \left(A_{652}-A_{750}\right)\\ -0.2538\times \left(A_{665}-A_{750}\right)+12.9508\left(A_{696}-A_{750}\right) \end{array}\right]}{\mathrm{Weight of the sample}} \end{array}$$

$$\mathrm{Total}\mathrm{Chl}\left(\mu g/g\right)=\frac{\left[\begin{array}{c} -22.0780\times \left(A_{630}-A_{750}\right)+10.2357\\ \times \left(A_{647}-A_{750}\right)-5.4224\times \left(A_{665}-A_{750}\right) \end{array}\right]}{\mathrm{Weight of the sample}}$$

$$\mathrm{Carotenoids}\left(\mu g/g\right)=\frac{\left[4-\left(A_{480}-A_{750}\right)\right]}{\mathrm{Wt}.\mathrm{of the sample}}$$

For the determination of fucoxanthin content “Fuc,” the fresh algal species was extracted in a mixture of dimethyl sulfoxide: water (4,1, v/v). The concentration of the extracted pigments was calculated using the following equation (19).


$$\begin{array}{l} Fucoxanthin\;\left(\mu g/g\right)\\ =\left[\begin{array}{l} 7.69-\left(A480-A750\right)-5.55-\\ \left[\begin{array}{l} \left(A631-A750\right)+\left(A582-A750\right)\\ -0.297-\left(A665-A750\right) \end{array}\right]\\ -0.377-\left(A665-A750\right) \end{array}\right]/Wt.ofsample. \end{array}$$

β-carotene and lycopene were determined in an acetone–hexane mixture according to Nagata and Yamashita (20), using the following formulas:


$$\begin{array}{c} \beta -Carotene\;\left(mg/100g\right)=0.216\times A663-0.304\\ \times A505+0.452\times A453. \end{array}$$

$$\begin{array}{c} \mathrm{Lycopene}\;\left(\mathrm{mg}/100g\right)=\left(-0.0458\times A663\right)+186\\ \times \left(0.372\times A505\right)-\left(0.0806\times A453\right). \end{array}$$

The vitamin C content of the tested algae was determined following the method of Pantelidis et al. (21). The results were expressed as mg ascorbic acid (AsA) per 100 g fresh weight (FW).

Vitamin E content was estimated in accordance with the method of Prieto et al. (22) and expressed as mg α-tocopherol equivalents per g of extract.

Total phenolic contents (TPCs) were measured using a colorimetric approach with the Folin–Ciocalteu technique (23). The absorbance of the reaction mixture was measured at 750 nm. The findings were presented in milligrams (+) gallic acid equivalents per g of seaweed dry weight (mg GAE/g DW). Total flavonoids were determined using a colorimetric method with aluminum chloride (24). The absorbance of the reaction mixture was estimated at 415 nm. The results were represented as mg (+) catechin equivalents (CE) per g of seaweed dry weight (mg CE/g DW). The tannin content was measured using a Folin–Ciocalteu reagent at 760 nm (25) and expressed as mg GAE/g DW.

### Antioxidant activity assay

Three methods were used to evaluate the antioxidant capacity of the methanolic extracts of brown species (100 μg/mL), including the total antioxidant capacity (TAC), 2,2-diphenyl-1-picrylhydrazyl (DPPH) free radical scavenging activity, and hydrogen peroxide assay.

The TAC of the methanolic algal extracts was determined in accordance with the method of Prieto et al. (22). Ascorbic acid was used as the standard. The TAC was expressed as ascorbic acid equivalents (AAE)/g.

The DPPH radical scavenging activity was determined according to the Yepez et al. (26) method. The DPPH free radical scavenging activity was calculated using the following equation:


$$\mathrm{DPPH scavenger activity}\;\left(\%\right)=\left[\left(A_{C}-A_{S}\right)/A_{C}\right]\times 100.$$

The hydrogen peroxide activity was estimated using the spectrophotometric method of Gülçin (27). AsA was used as a standard. The percentage of algal sample scavenging activity was calculated using the formula:


$$\mathrm{Free radical scavenging}\;\left(H_{2}O_{2}\right)\;\left(\%\right)=\left[\left(A_{C}-A_{S}\right)/A_{C}\right]\times 100.$$

### Statistical analysis

Each experiment was carried out in triplicate, and the results are presented as the means and standard deviation. The statistically significant difference between the studied seaweed parameters was detected using one-way ANOVA. The correlation matrix (*r*) was performed using IBM-SPSS version 20 at a significance level of *p* ≤ 0.05 to determine the relationship among different estimated parameters.

## Results and discussion

### Proximate composition

**Figure 3:**
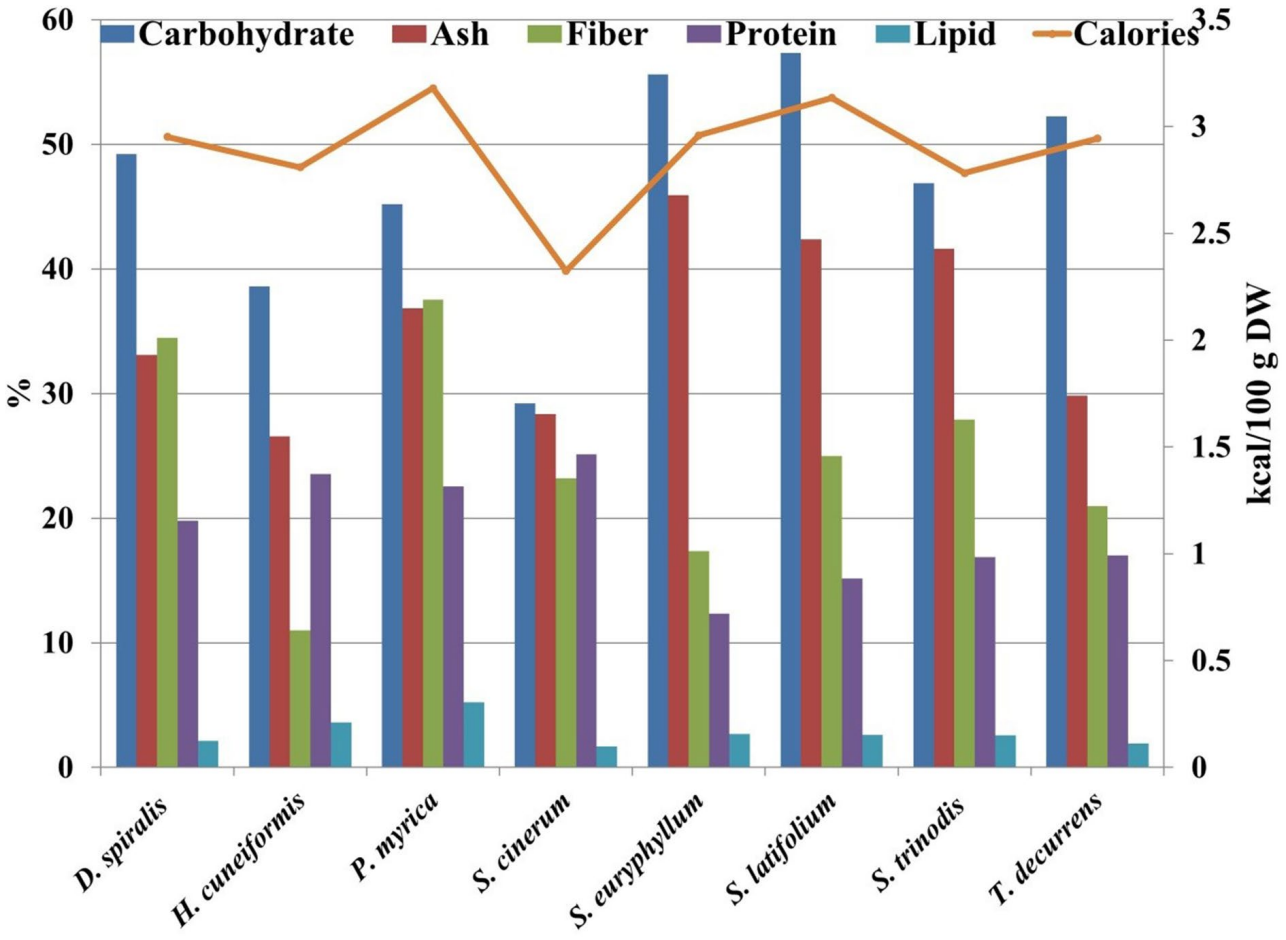
Nutrional contents ratio of the tested species.

The cell walls of brown algae are characterized by a fibrillar compartment consisting mainly of cellulose microfibrils embedded in an amorphous matrix of acidic polysaccharides bound together by proteins (28). Carbohydrates are the main components that influence different physiological responses in genes regulated during photosynthesis, metabolism, and self-protective replications. As illustrated in Figure 3 and Table 1, carbohydrate content is the main compound compared with protein and lipid contents.

**Table 1:** Pigment contents of the collected brown macroalgae.

Seaweed species	Chl *a* μg/g FW	Chl *c* μg/g FW	Total Chls μg/g FW	Carotenoid μg/g FW	Fucoxanthin μg/g FW	β-carotene mg/100 g FW	Lycopene mg/100 g FW
*Polycladia myrica*	254.5 ± 12.5	23.9 ± 3.2	556.99 ± 15.52	24.20 ± 0.9	3.12 ± 0.8	0.55 ± 0.01	0.48 ± 0.08
*Sirophysalis trinodis*	312.2 ± 1.3	25.9 ± 4.5	616.40 ± 18.23	27.40 ± 1.1	2.65 ± 0.2	0.49 ± 0.00	0.129 ± 0.02
*Dictyota spiralis*	356.8 ± 4.2	23.97 ± 1.2	594.43 ± 13.25	30.60 ± 2.1	2.84 ± 0.2	0.71 ± 0.03	1.613 ± 0.4
*Hormophysa cuneiformis*	231.7 ± 4.5	22.5 ± 2.5	363.52 ± 14.52	26.1 ± 1.5	2.49 ± 0.4	0.46 ± 0.04	0.018 ± 0.002
*Sargassum cinerum*	201.1 ± 6.1	13.4 ± 2.5	353.67 ± 12.25	60.44 ± 2.5	3.55 ± 0.5	0.49 ± 0.01	0.04 ± 0.02
*Sargassum euryphyllum*	432.6 ± 2.5	5.4 ± 1.2	689.37 ± 22.13	37.40 ± 1.2	3.49 ± 0.1	0.52 ± 0.03	1.60 ± 0.2
*Sargassum latifolium*	301.3 ± 11.2	7.96 ± 1.3	654.9 ± 11.5	33.60 ± 2.2	1.31 ± 0.2	0.48 ± 0.04	0.09 ± 0.01
*Turbinaria decurrens*	355.4 ± 6.2	7.30 ± 1.8	543.9 ± 18.2	27.20 ± 1.2	0.97 ± 0.2	0.48 ± 0.02	0.40 ± 0.05
Min	201.1	5.36	353.67	24.20	0.97	0.46	2.90
Max	432.6	25.87	689.37	60.44	3.55	0.71	411.12
F value	399.9*	42.8*	4.5*	50.5*	13.7*	68.4*	63.64*

Results are expressed as means of three replicates ± standard deviations SD. An *F* value * means that the difference is significant at the *p* ≤ 0.05 level.

The results showed that the carbohydrate content of the collected species ranged from 29.22% DW in *S. cinerum* to 57.32% DW in *S. latifolium*. These results are similar to those of brown species collected from Hurghada City during spring reported by Fouda et al. (29) (24.79–41.66% DW) and Farghl et al. (30) (27.5–38.62% DW). The variation in algal carbohydrate content may be due to algal species, growth stage, habitat, metabolic preferences, and photosynthetic activity (31). In the present work, *S. cinerum* achieved the maximum protein content with 25.13% DW, and the minimum content was observed in *S. euryphyllum* with 12.34% DW. The protein content in all studied seaweeds (12.34–25.13% DW) was greater than that in traditional protein sources, such as milk (3.4%) (32) or eggs (12.1%) (33). Protein data exhibited a higher ratio than brown algae collected from the Red Sea, as estimated by Farghl et al. (30) (5–17% DW) and Fouda et al. (29) (2.81–5.31% DW). Brown seaweeds commonly have protein contents between 3 and 15% DW. However, some species contain up to 24% protein (34). Therefore, brown seaweed-derived protein can be a source of protein that is sustainable and highly nutritious and does not require land for cultivation or fresh water for irrigation (6). The differences in the protein content of seaweeds are due to species, different habitats, time of the year, and levels of maturity (31). At present, there are few products on the functional food market that utilize seaweed proteins, most of which are used in human foods and animal feeds because of their antioxidant properties (35).

The total lipid content of brown species varied between 1.67% DW in *S. cinerum* and 5.21% DW in *P. myrica* (Figure 3). However, these values were higher than previous results of brown algae collected from the Red Sea (29) (0.11–0.27% DW) or (1.12–3.04% DW) (30). The differences in lipid contents were assigned to either environmental parameters or species types or both (31).

In general, seaweeds are characterized by their high fiber content (36). Most of the dietary fiber provided by seaweed cannot be absorbed by the human body, which causes the caloric intake of seaweed to be low. In addition, the soluble fiber contained in algae, once it reaches the human intestine, forms a viscous mass that traps digestive enzymes and some other nutrients, which slows down the digestibility of food and decreases the absorption of nutrients in the intestine (37). In the present work, the fiber content ranged from 11.01% DW in *H. cuneiformis* to 37.53% DW in *P. myrica* (Figure 1). These results are consistent with a previous work (30), who detected that brown seaweed fiber content varied between 11.63% DW and 35.65% DW. Moreover, a recent work reported that *P. myrica* has a higher content of dietary fiber (32.39 mg/g) than other seaweeds from the Southern Red Sea, Eritrea (38).

Seaweeds contain considerably higher concentrations of all necessary minerals than any other land vegetation, which is represented by their ash content (38). The highest ash content of the seaweed species tested was found in *S. euryphyllum* (45.91% DW), whereas *H. cuneiformis* showed the lowest ash content (26.57% DW) (Figure 3). Ash content varies with environmental, geographical and physiological factors (31). Ash contents were compatible with previous works found in seaweeds collected from the Red Sea (19.6–45.48% DW) (29) and from the Southern Red Sea (30.30–50.91%) (38). Based on the biochemical results of the selected species, *P. myrica* can be labeled as an alternative protein, and a high content of lipids (5.21% DW) and fiber (37.53% DW) could contribute to a sustainable and nutritious diet.

The estimated calorie value of the tested seaweeds was low, ranging between 2.32 and 3.52 kcal/100 g. A similar finding was obtained by El Zokm et al. (39), but these values were lower than those previously estimated (137.47–167.51 kcal/100 g) (40). However, low calorie values may lead to high quantities of carbohydrate and protein and low lipid contents in algal species. Consequently, these algae can be used as supplements and alternative sources to lower the risk of obesity.

### Pigments content (chlorophylls, β-carotene, fucoxanthin, and lycopene)

The specific photosynthetic pigments and their concentrations in brown seaweeds vary depending on the environmental conditions, including season, light intensity, growth depth, and extracted solvent (19, 41). Additionally, the species and morphological structure could affect pigment concentrations (42). Information on the pigment composition and quantity of brown seaweeds may promote the selection of pigment sources for future use. The chlorophyll *a* and *c* contents of the selected species had the following ranges: 201.1–432.60 μg/g FW, 106.29–344.80 μg/g FW, and 5.36–25.87 μg/g FW. The total chlorophyll content fluctuated from 353.67 to 689.37 μg/g FW in *S. cinerum* and *S. euryphyllum*, respectively. These results indicated that *S. euryphyllum* is a potential source of natural pigments in brown seaweed. In accordance with our results, Garcia-Perez et al. (41) reported that the total chlorophyll content of brown seaweeds varied between 415.3 μg/g and 1555.2 μg/g FW. In addition, Ismail et al. (3) reported an average total chlorophyll content of 522 μg/g in brown seaweed.

Chlorophyll *c* in brown seaweed plays an important role in light harvesting antennae. In the seaweed species investigated, the chlorophyll *c* content in *S. trinodis* (25.87 μg/g FW) was higher than that in the other tested seaweeds. Osório et al. (19) reported 15.98 ± 1.6 and 17.9 ± 0.7 μg/g chlorophyll *c* content in the brown algae *Undaria pinnatifida* and *Laminaria ochroleuca*, respectively, which supports the obtained results. Therefore, conducting considerable research on pigment contents in algae is necessary in the future because of the significant demand for natural pigments in food products, particularly dairy, and beverages as food additives, and in cosmetics.

Carotene is a primary source of vitamin A, which can affect many different tissue types. It is also essential for retinal function (43). Carotenoids have 750 known natural sources, approximately 200 of which are collected from algal sources, and many of these are only found in a limited number of species (44). Carotenoids may participate in antioxidant activities via the transfer of the excess energy of singlet oxygen (O) in the long central allenic chain (44).

Carotenoids can scavenge oxidizing free radicals via three primary reactions: (i) electron transfer (ET) between the free radical (R·) and Crt, resulting in the formation of a Crt radical cation (Crt·+) [Equation (1)] or Crt radical anion (Crt·−) [Equation (2)]; (II) they can transfer the electrons forming a radical cation (RCrt·+) [Equation (3)]; (III) hydrogen atom transfer leading to a neutral Crt radical (Crt·) [Equation (4)] (45).

(1) $$R\cdot +\mathrm{Crt}\to R^{-}+\mathrm{Crt}\cdot^{+}$$

(2) $$R\cdot +\mathrm{Crt}\to R^{+}+\mathrm{Crt}\cdot^{-}$$

(3) $$R\cdot +\mathrm{Crt}\to \mathrm{RCrt}\cdot^{+}$$

(4) R⋅+Crt→RH+Crt⋅

In the present research, the carotenoid content ranged from 24.2 μg/g in *P. myrica* to 60.44 μg/g in *S. cinerum*. The range of the estimated carotenoids was higher than that recorded in the brown algae *S. polycystum* (45.28 ± 1.77 μg/100 g DW) collected from the coast of Peninsular Malaysia by Fiedor (45), but it was lower than that estimated by Heriyanto et al. (42) (0.55 to 4.06 mg/g DW).

β-Carotene, fucoxanthin, and lycopene are obtained from the main carotenoids present in algae. β-Carotene is a liposoluble pigment that has pro-vitamin A activity with a long chain of conjugated double bonds produced from an acyclic structure. In the present investigation, the average content of β-carotene (0.52 ± 0.08 mg/100 g DW) is in accordance with that reported by De Sousa (46), with an average content of 0.4185 ± 0.1559 mg/100 g DW for brown algae *Lobophora* collected from Brazil. This result was lower than that recorded from the Red Sea (0.47 mg/g DW) (29) or 0.55 to 4.06 mg/g DW obtained by another work (42).

Fucoxanthin is a distinctive pigment present in brown algae, and it is a major accessory pigment found in chloroplasts, together with chlorophyll *a* and *c* and β-carotene. It masks the other pigments, like chlorophyll *a*, *c*, β carotenes, and other xanthophylls (5). In addition, it is an abundant carotenoid in nature, and it constitutes 10% of total carotenoid production (19). Fucoxanthin has large application potential as an antioxidant, anticancer, antidiabetic, antiobesity, and anti-inflammatory agent *in vitro* and *in vivo* (5, 47). Fucoxanthin exerts its antioxidant action by donating an electron to the reactive oxygen species (ROS) instead of a proton (hydrogen), as do most antioxidants, such as AsA or β-carotene. Fucoxanthin can also act on the ROS under physiological conditions of hypoxia compared to most antioxidants present in foods (24).The unique chemical structure of fucoxanthin has an allenic bond and an acetyl functional group, which are responsible for its antioxidant properties (48). Using chemiluminescence detection, Nishida et al. (49) reported that fucoxanthin had higher singlet oxygen-buffering activity than vitamin C, vitamin E, and lycopene. The fucoxanthin content ranged between 0.96 and 3.55 μg/g FW in *T. decurrens* and *S. cinerum,* respectively (Table 1). *Sargassum* species had the highest fucoxanthin content compared with other species. In general, Sargassaceae species had higher abundances than species from other groups (42).

Lycopene is an acyclic form of β-carotene without provitamin. It is a potent antioxidant agent, showing twice the activity of β-carotene in quenching singlet oxygen and with ten times more antioxidant activity than α-tocopherol *in vitro* (47). Its antioxidant effect is enhanced by the fact that it is unstable and highly reactive to oxygen and free radicals. The main difference between β-carotene and lycopene is that β-carotene is the main precursor of dietary vitamin A, whereas lycopene has no pro-vitamin A activity. Lycopene acts mainly as an antioxidant agent by activating the Nrf2/ARE transcription pathway to alleviate oxidative stress (47). Based on several clinical and epidemiological studies conducted in many populations, natural carotenoids (lutein, β-carotene, and lycopene) are beneficial to human and animal health (50). For the first time, lycopene was estimated in Egyptian seaweeds, ranging from 0.02 to 1.61 mg/100 g with an average value of 0.55 mg/100 g. Similarly, Garcia-Perez et al. (41) detected the lycopene content of different macroalgae ranging from 0.32 to 11.2 mg/100 g DW. To our knowledge, no research has indicated the existence of lycopene in the selected algal species.

### Vitamin content

The vitamin profile of seaweeds is influenced by a number of factors, including seaweed species, developmental stage, geographical location, salinity, seasons, light availability, and seawater temperature (51). Vitamins with a strong antioxidant capacity can be used as the first-line of therapeutic defense against cancer before pharmacological cancer treatment (11, 52).

**Figure 4:**
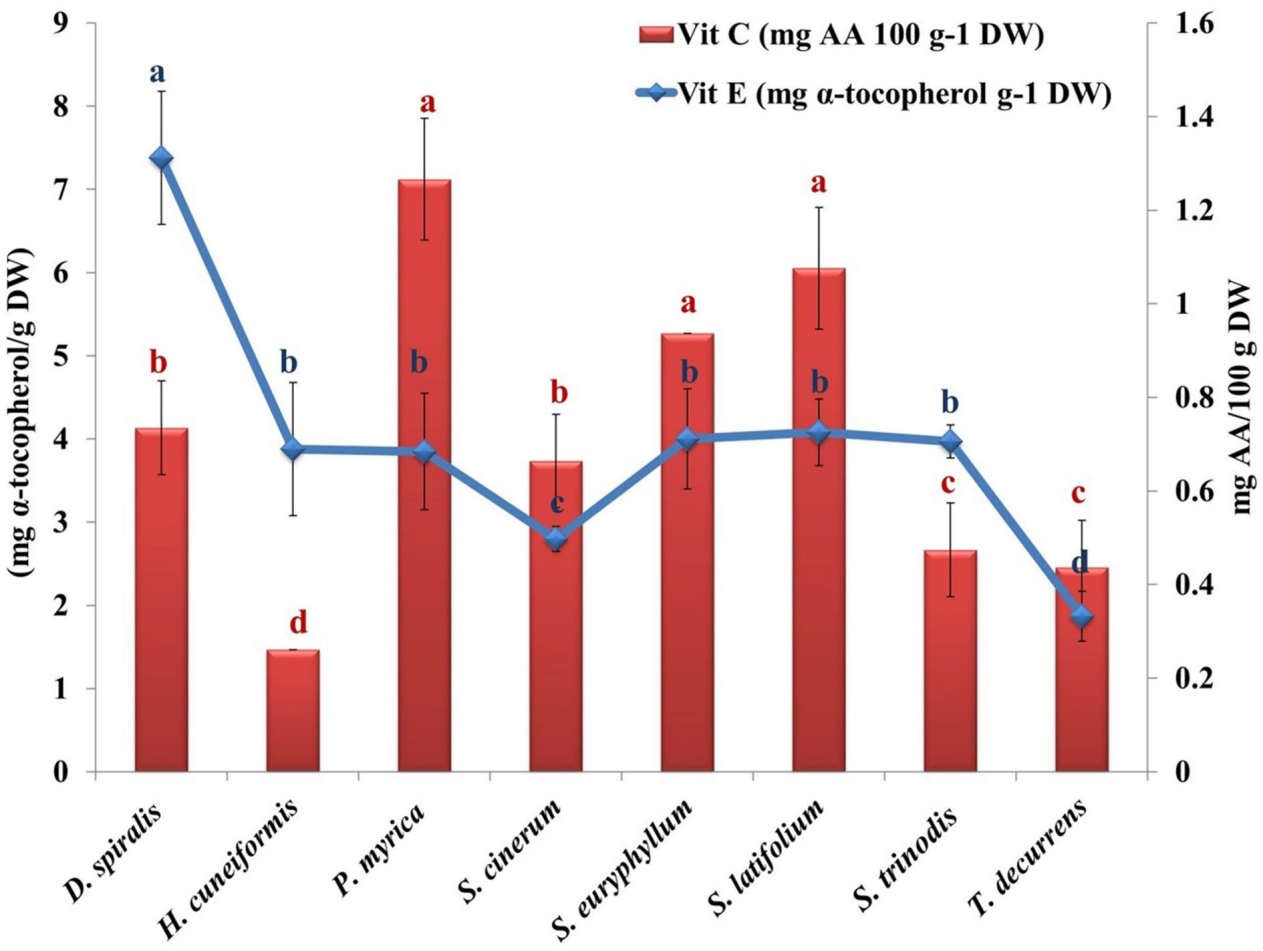
Variation in algal vitamins (C & E) contents of the tested species. Values are means ± SD of three replicates. Similar superscript letters indicate insignificant difference between species at *p* ≤ 0.05 level of significance.

Vitamin C is an essential nutrient required to maintain normal physiological functions in animal cells. Some animals cannot synthesize it, including invertebrates and fishes, because of the lack of L-gulonolactone oxidase, which catalyzes the terminal step in the conversion of glucose to AsA (53). In the current work, a higher value of vitamin C was found in *P. myrica* (1.27 mg AA/g DW) than in the other seaweeds investigated. A lower value (0.261 mg AA/g DW) was obtained for *H. cuneiformis* (Figure 4). These values are higher than those of other reported brown seaweeds from the Red Sea, such as *Colpomenia sinuosa* (0.0145 μg/g DW) (30). AsA has shown cancer preventive activity by neutralizing free radicals before they can damage DNA and thus initiate tumor cell growth. In addition, it can also act as a prooxidant agent, helping the body’s own free radicals to destroy tumors in their early stages (52).

Vitamin E is an important fat-soluble antioxidant that prevents oxidation of polyunsaturated fatty acids absorbed from the diet. The α-tocopherols block the production of ROS formed during oxidation and inhibit the oxidation of low-density lipoproteins (11). In addition, α-tocopherol donates its phenolic hydrogen atom to a peroxyl radical and converts it to a hydroperoxide. The tocopheroyl radical formed is sufficiently stable and cannot continue the chain. It is therefore removed from the cycle by reacting with another peroxyl radical to form an inactive, non-radical product. The estimated vitamin E ranged from 1.87 mg/g DW in *T. decurrens* to 7.38 mg/g DW in *D. spiualis* (Figure 4). The average values of vitamin E in the selected samples were similar to those of *Macrocystis pyrifera*, which was approximately 1.33 mg/g DW (54). However, this value was higher than that estimated for the brown algae *Ascophyllum* and *Fucus* sp., which contained 0.2 and 0.6 mg/g DW of vitamin E, respectively (35).

### Phenolic compounds

Polyphenols contained in seaweeds represent a vast number of secondary metabolites, including approximately 8.000 natural compounds that possess vital biological functions, including antioxidant and free radical scavenging capacity (3, 9, 10). The content of phenolic and flavonoid compounds is high in algae grown under extreme climatic conditions, indicating their key role in chelating metal ions, decreasing radical generation, and strengthening the internal antioxidant system. These functions contribute to the prevention of diseases caused by the ROS. In addition, some of the polyphenols contained in marine algae are metabolites not found in terrestrial plants, which makes their contribution through algae complementary to that of terrestrial vegetables (55).

**Figure 5:**
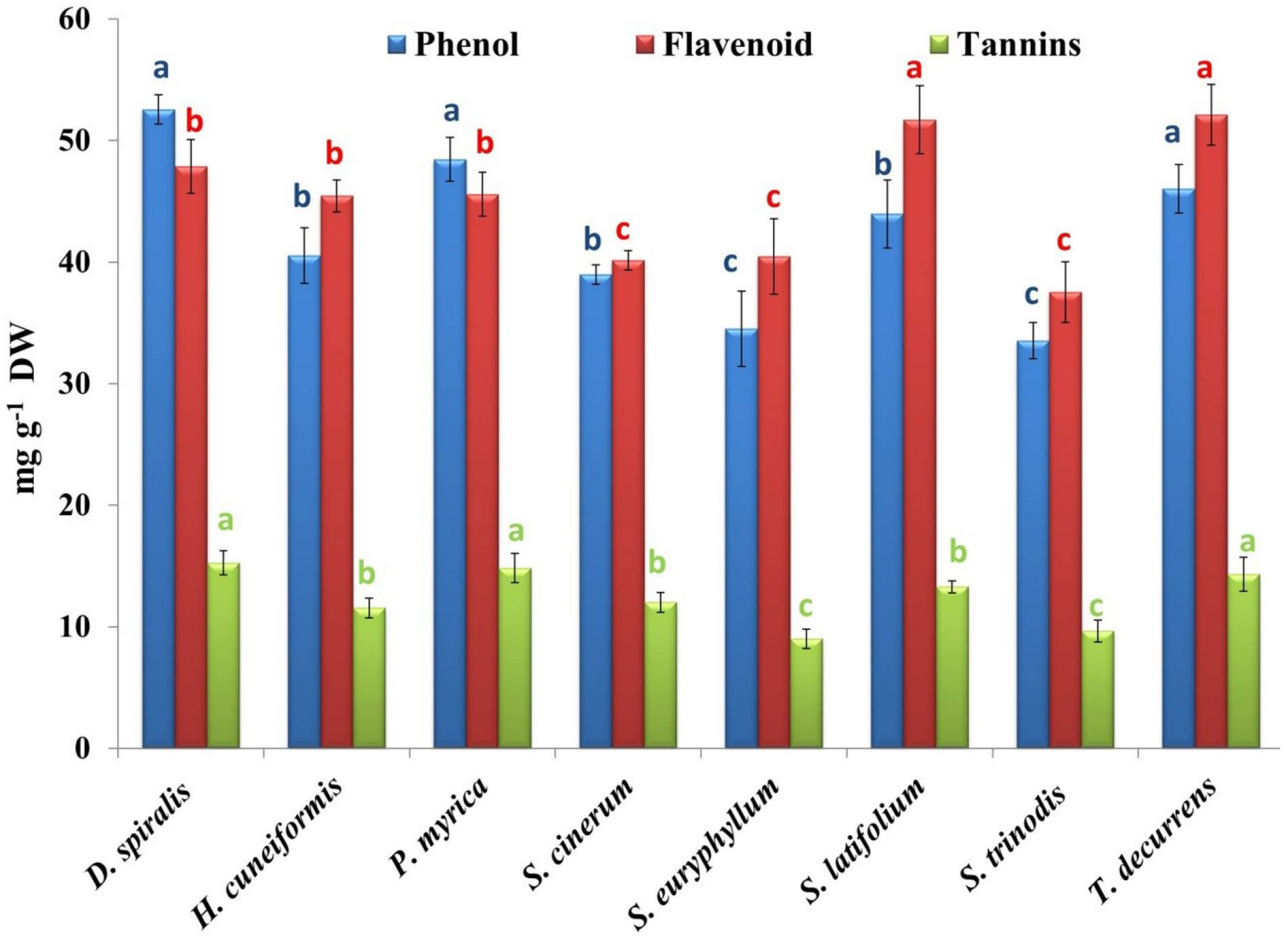
Phytochemical contents of phenols, flavonoids, and tannins in the tested seaweeds. Values are means ± SD of three replicates. Similar superscript letters indicate insignificant difference between species at *p* ≤ 0.05 level of significance.

Brown seaweeds are characterized by the production of phenols and flavonoids (56). In this study, three phenolic compounds were evaluated, including phenol, flavonoids, and tannins (Figure 5). *D. spiralis* contained the highest total phenolic content (TPC) (52.56 mg GAE/g DW). However, *S. trinodis* had the lowest TPC (33.54 mg GAE/g DW). Several studies have reported comparable phenol levels (41.82 ± 0.91 mg GAE/g DW) in brown algae compared with our findings (2). The phenolic concentration in the tested algal methanolic extracts exceeded that reported by Elkhateeb et al. (57) in the ethanolic extract of *S. subrepandum* collected from Hurghada (2.88 ± 0.22 mg GAE/g DW); however, the estimated TPC was lower than that (29.26 ± 0.01 mg GAE/g DW) recorded in *Colpmenia sinus* by Shobier et al. (58) and by Farghl et al. (30) (14.14 ± 0.1 mg GAE/g DW) in *T. ornate*. This variation in total phenol content might be related to the types of algal species and the solvent used for extraction. The DPPH activity was evidenced in the present work by an equation that seems to depend mainly on the phenol content.


$$\mathrm{DPPH}=0.848\;\mathrm{Phenol}+37.69\;R^{2}{}_{=}\;0.937.$$

Flavonoids consist of a large group of polyphenolic compounds with a benzo-𝛾-pyrone structure and antioxidant activity, which depends on the number and position of the free OH groups (59, 60). Flavonoids stabilize the ROS by reacting with reactive radical compounds. The hydroxyl group of flavonoids had the high reactivity to convert free radicals to inactive form as shown in the following equation (55):


Flavonoid(OH)+R•>flavonoid(O•)+RH.

where R • is a free radical and O • is an oxygen free radical.

The present study achieved a total flavonoid content (TFC) of the tested seaweeds ranging from 37.54 to 52.11 mg CAE/g DW, which was higher than that estimated by Elkhateeb et al. (57) (3.54 ± 0.59 mg CAE/g) and Farghl et al. (30) (9.98 ± 0.09 mg CAE/g DW) in different macroalgal species harvested from the Red Sea.

Tannins can chelate metal ions such as Fe(II) and interfere with one of the reaction steps in the Fenton reaction, hydrogen peroxide (H_2_O_2_), and ferric ions (Fe^2+^), thereby hindering oxidation. The inhibition of lipid peroxidation by tannin constituents can act via the inhibition of cyclooxygenase (56). The highest and lowest contents of tannins were found in *D. spiralis* (15.26 mg GAE/g DW) and *S. euryphyllum* (9.01 mg GAE/g DW), respectively (Figure 5). The results obtained were smaller than those detected by Shobier et al. (58), who reported that the total tannin of the brown alga *C. sinuosa* fluctuated from 0.14 to 4.55 mg/g DW, depending on the extracted solvents. Moreover, Ismail et al. (3) stated that the total tannin content in brown algae collected from the Mediterranean Sea ranged from 1.7 to 4.67 mg/g DW.

### Antioxidant activity

The antioxidant activity of seaweeds arises from their chemical compositions, including their phenolic compounds, pigments, polysaccharides, vitamins, and precursor contents (such as AsA), micro- and macroelements, and proteins (61). Antioxidants provided by seaweeds could serve as free radical scavengers and mitigate the ROS/free radicals or could contribute to preventing the formation of hydroxyl radicals by deactivating free metal ions through chelation or converting H_2_O_2_ to other innocuous compounds (such as water and oxygen) (62). Among the different types of algae, it is a well-known fact that brown algae usually have the highest antioxidant activity, followed by red and green algae (63).

**Figure 6:**
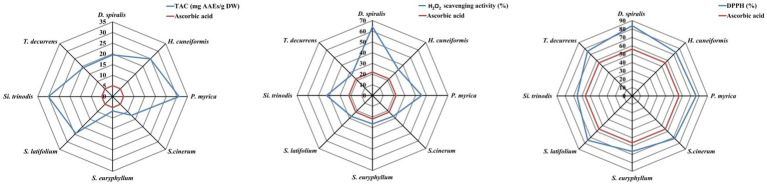
Antioxidant activity assays of the selected brown species.

Three simple methods were used to evaluate the antioxidant capacity of the methanolic extract of the brown species, including the DPPH free-radical scavenging activity, total antioxidant capacity and hydrogen peroxide assay (Figure 6; Table 2). The maximum DPPH scavenging activity of 83.97% inhibition was observed in *D. spiralis,* and the minimum of 65.78% was observed in *S. trinodis*. These results are better than the AsA standard (62.35%). These findings are consistent with those of Farghl et al. (30) in brown algae harvested from the Red Sea with a maximum inhibition of 72.48%. In contrast, the TAC approach may be broadly classified as ET, which was used to assess and evaluate the antioxidant activity of various seaweed components (electron donors). TAC showed that the scavenging value fluctuated from 6.71 mg AAE/g DW for *S. euryphyllum* to 31.05 mg AAE/g DW for *S. cinerum*. The other six species exceeded that reported by Farghl et al. (30), who recorded the maximum TAC of brown seaweeds (17.41 mg AAE/g DW). The highest and lowest H_2_O_2_ scavenging activities among the studied seaweeds were observed in *D. spiralis* (64.10%) and *S. euryphyllum* (26.53%), respectively. In general, this estimated H_2_O_2_ scavenging activity value of the tested algae was significantly higher than that of the positive control “AsA” (21.9 ± 1.12%; Figure 6). Ismail et al. (65) demonstrated that the antioxidant activity of *S. aquifolium* and *S. euryphyllum* collected from the Red Sea may be related to their polysaccharide content.

**Table 2:** Comparative studies of antioxidant activity with different brown species.

Species	Habitat	Antioxidant assay			Reference
		TAC (mg/g ASA)	H_2_O_2_ (%)	DPPH (%)	
The tested brown species	M.S.	6.71–31.05	26.53–64.10	30.09–65.25	This work
*S. wightii*, *P. boryana*		0.42 ± 0.19			Ismail et al. (3)
*Cy. myrica, D. spiralis, S. euryphyllum*, *T. decurrens*	R.S.		15.48–33.18	56.87–64.88	Ismail et al. (64)
*P. pavonica*, *Ptatelonia fascia*, *Sargassum vulgare*	M.S.	1.903–2.354			El Zokm et al. (39)
*Turbinaria ornata, Polycladia indica*	R.S.	15–16		60.5–70.5	Farghl et al. (30)
*Padina boergesnii*, *Sargassum subrepandum* M Alam, *S. cinerum* M Alam, *S. aquifolium*	R.S.		0.33–2.72	5.4–22.81	Elkhateeb et al. (57)

M.S., Mediterranean Sea; R.S.; Red Sea.

### Correlation analysis

Table 3 shows the statistically significant correlation between the three antioxidant activities of the selected species and 19 estimated parameters. Significant correlations were observed between phenol, DPPH (*r* = 0.967), and H_2_O_2_ activity (*r* = 0.564), which confirmed that the total phenol content is closely linked to these antioxidant activities. Moreover, vitamin E and β-carotene exhibited a positive relationship with DPPH (*r* = 0.507; *r* = 0.67) and H_2_O_2_ activity (*r* = 0.832; *r* = 0.855), respectively_._ Furthermore, a significant relationship was found between flavonoids (*r* = 0.699) and tannins (*r* = 0.921) and DPPH efficiency. Positive correlations were found between Chl *c*, TAC (*r* = 0.675), and DPPH ability (*r* = 0.767); between lipids and TAC (*r* = 0.577); and between fiber and H_2_O_2_ ability (*r* = 0.69). This statistical analysis confirmed the vital role of different estimated antioxidants with different antioxidant mechanisms as previously illustrated.

**Table 3:** Statistically significant correlations between the estimated antioxidants activity and biochemical composition in the eight studied seaweeds at *p* ≤ 0.05.

TAC	Chl c (*r* = 0.675); Lipid (*r* = 0.577).
DPPH	β-carotene (*r* = 0.670); Vitamin E (*r* = 0.507); Phenol (*r* = 0.967); Flavonoid (*r* = 0.699); Tannins (*r* = 0.921).
H_2_O_2_	Chl c (*r* = 0.767); β-carotene (*r* = 0.855); Vitamin E (*r* = 0.832); Phenol (*r* = 0.564); Fiber (*r* = 0.69).

## Conclusion

Seaweeds contain a variety of bioactive compounds that have nutritional and therapeutic attributes, making them an intriguing potential resource. They contain low-calorie content and a variety of important nutrients, such as proteins, vitamins, and fiber, as well as the presence of bioactive substances with functional qualities that provide further benefits for health, such as phenolic compounds. There is a growing trend to use Phaeophyta in functional food production around the world and holds great promise for the future. Seaweeds are high in a variety of nutritious compounds as well as unique metabolic chemicals (phlorotannins, fucoxanthin, and sulfated polysaccharides) with intriguing bioactivities, making them excellent candidates for nutraceutical applications with higher value-added.

So, this study has an innovative approach to evaluating the value of the studied brown seaweed as a substitute food source. Applying inexpensive and simple laboratory techniques for analyzing seventeen components is very encouraging. Based on energy and calorie contents, integrating a considerable amount of seaweeds into the diet can reduce the appetite for further eating. In particular, the phenolic compounds are effective antioxidants in marine algae. Moreover, algal vitamins can be used as safe nutraceutical substances. The production of seaweed-based functional food at an industrial scale could address the limitations and challenges faced by the manufacturer. Unfortunately, there remain considerable challenges in quantifying these benefits, as well as possible adverse effects. First, there is a limited understanding of the nutritional content of various algal species, geographical regions, and seasons, all of which can substantially affect their dietary value. The second issue is quantifying which fractions of algal foods are bioavailable to humans and which factors influence how food constituents are released, ranging from food preparation through effects in the gut microbiome. The third understands how algal nutritional and functional constituents interact in human metabolism. Recently, research has been done on a wide range of food products with incorporated macroalgae, as well as some marketed macroalgae-based food products commercially available today, like food ingredients, nutraceuticals, food supplements, and food hydrocolloids.

## Data availability statement

The original contributions presented in the study are included in the article/Supplementary material, further inquiries can be directed to the corresponding author.

## Author contributions

MI: conceptualization, collection, identification of seaweed, data processing, writing original draft, and writing review. GE: conceptualization, methodology, data processing, formal analysis, writing original draft, and writing review. JM: formal analysis, reviewing original draft, and formatting. All authors contributed to the article and approved the submitted version.

## Funding

This research did not receive any specific grant from funding agencies in the public, commercial, or not-for-profit sectors.

## Conflict of interest

The authors declare that the research was conducted in the absence of any commercial or financial relationships that could be construed as a potential conflict of interest.

## Publisher’s note

All claims expressed in this article are solely those of the authors and do not necessarily represent those of their affiliated organizations, or those of the publisher, the editors and the reviewers. Any product that may be evaluated in this article, or claim that may be made by its manufacturer, is not guaranteed or endorsed by the publisher.
