# Supplementary material for: Nutritional, bioactive compounds content, and antioxidant activity of brown seaweeds from the Red Sea
Source: Front Nutr. 2023 Jul 26;10:1210934. doi: 10.3389/fnut.2023.1210934 (PMC10410277; doi:10.3389/fnut.2023.1210934)
Supplement: Supplementary file 1 [file Data_Sheet_1.pdf]

Table S1. The nutrional contents of the selected seaweed species

Algal spp.	Carbohydrate %	Protein %	Lipid %	Ash %	Fiber %	Calories (kcal 100 g-1 DW)
D. spiralis	49.20	19.81	2.13	33.1	34.458	2.952
H. cuneiformis	38.58	23.54	3.60	26.570	11.008	2.809
P. myrica	45.20	22.54	5.21	36.84	37.534	3.179
S. cinerum	29.22	25.13	1.67	28.360	23.202	2.324
S. euryphyllum	55.60	12.34	2.69	45.91	17.34	2.959
S. latifolium	57.32	15.15	2.61	42.380	24.988	3.134
S. trinodis	46.89	16.89	2.57	41.610	27.922	2.782
T. decurrens	52.25	17	1.93	29.82	20.962	2.944
