# Supplementary figures and images for: Nutritional, bioactive compounds content, and antioxidant activity of brown seaweeds from the Red Sea
Source: Front Nutr. 2023 Jul 26;10:1210934. doi: 10.3389/fnut.2023.1210934 (PMC10410277; doi:10.3389/fnut.2023.1210934)

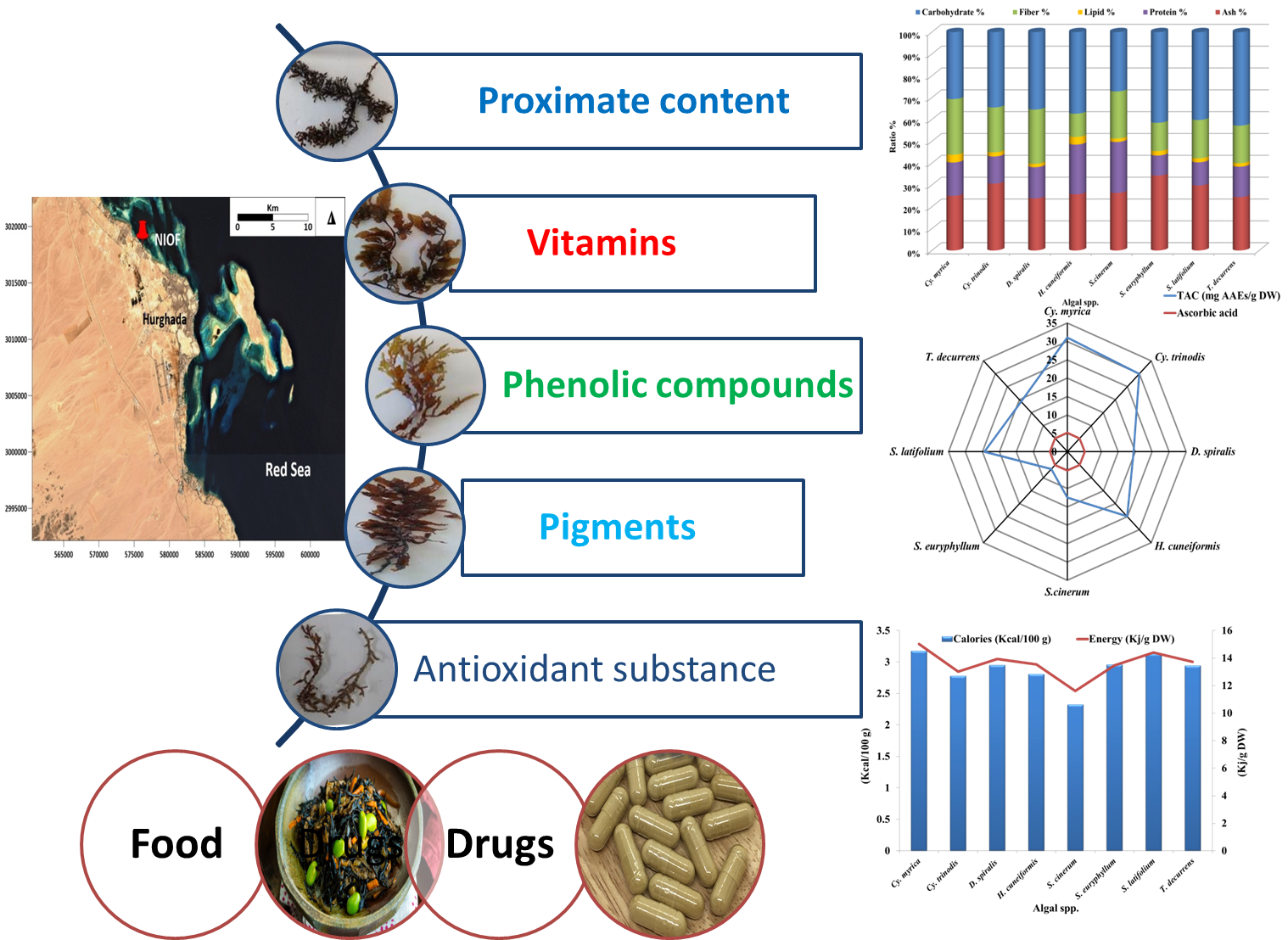

Supplement: Supplementary file 2 [file Image_1.PNG]
